# Understanding implicit and explicit learning in adolescents with and without anorexia nervosa

**DOI:** 10.1186/s40337-021-00431-7

**Published:** 2021-06-29

**Authors:** Lot C. Sternheim, Miriam I. Wickham, Unna N. Danner, Todd W. Maddox, Vincent J. Filoteo, Megan E. Shott, Guido K. W. Frank

**Affiliations:** 1 Department of Clinical Psychology, Universiteit Utrecht, Heidelberglaan 1, 3508 TC Utrecht, The Netherlands; 2 Utrecht, The Netherlands; 3 Department of Social Health and Organisation Psychology, Universiteit Utrecht, Heidelberglaan 1, 3508 TC Utrecht, The Netherlands; 4 Altrecht Eating Disorders Rintveld, Wenshoek 4, 3705 WE Zeist, The Netherlands; 5 Department of Psychology, University of Texas, Austin, USA; 6 Department of Psychiatry, University of California San Diego, La Jolla, CA USA; 7 Eating Disorders Center for Treatment and Research, University of California San Diego, San Diego, CA USA

**Keywords:** Anorexia nervosa, Adolescents, Implicit learning, Explicit learning

## Abstract

**Background:**

Cognitive disturbances such as impairments in learning are thought to play a role in adult Anorexia Nervosa (AN). It is remains unclear to what extent these disturbances result from starvation of the brain, or relate to an abnormal premorbid cognitive profile. This study investigates learning processes in adolescents with AN, hypothesizing that implicit learning is intact, as found previously in explicit learning tasks. Secondly, we hypothesized that anxiety and depression symptoms, inherent to AN, are associated to learning processes in AN.

**Methods:**

In total 46 adolescents diagnosed with AN and 44 control participants were administered an implicit category learning task in which they were asked to categorize simple perceptual stimuli (Gabor patches) based on a linear integration (i.e., an implicit task) of orientation and spatial frequency of the stimulus. A subgroup of adolescents (*n* = 38) also completed a task assessing explicit learning.

**Results:**

Model-based analyses indicated that adolescents with AN performed significantly more accurately compared to their healthy peers regardless of whether they used the optimal strategy or not. Depression and anxiety did not relate to learning performance in the AN group.

**Conclusions:**

Overall, our findings of augmented implicit and explicit learning in adolescents with AN corroborate recent studies that suggested higher stimulus-response learning during prediction error paradigms. Learning disturbances in adult AN may then be at least partly due to long-term malnourishment, highlighting the importance of early recognition and refeeding in treatments for AN.

## Plain English summary

We know that some adults with anorexia nervosa (AN) experience difficulties in learning processes. It is remains unclear to what extent these difficulties result from long-term starvation of the brain. This study looked at learning processes in adolescents with AN who have a relatively short duration of illness. We also investigated whether anxiety and depression affected learning. Forty-six adolescents diagnosed with AN and 44 control participants completed tasks that assessed learning and questionnaires assessing depression and anxiety. We found that adolescents with AN performed more accurately compared to their healthy peers. However, depression and anxiety did not relate to learning performance. Overall, our findings suggest that individuals with AN, relative to their healthy peers, may be quicker in forming automatic responses and behaviors to cues. Learning disturbances in adult AN may then be at least partly due to long-term malnourishment, which highlights the importance of early recognition of AN and refeeding in treatments for AN.

## Background

Anorexia nervosa (AN) is a severe psychiatric disorder with the highest mortality rates across mental disorders [1]. AN is characterized by extreme restriction of intake or purging of food, fear of gaining weight and disturbed experience of body weight or shape (DSM-5, [2]). The lifetime prevalence of AN among women is up to 4% [3] with a crude mortality rate of approximately 5% per decade [1, 2]. This debilitating disorder most typically develops during adolescence or young adulthood [4] and research suggests that prepubertal and early adolescent onset of AN may be on the rise [5].

Whilst available treatments for adolescents, in particular family-based interventions, are associated with good rates of remission (approximately 70% [6];) there is still a subgroup of young people who do not benefit from this treatment. lLittle is known about factors contributing to a more chronic prognosis [6, 7].

Recently it has been suggested that the focus for AN treatment should shift from mainly treating physical symptoms (i.e weight loss), and psychiatric symptoms (i.e., depression), to potentially underlying pathologies, such as disturbed cognitive processes, which have been described in adults with AN [8]. To further advance this direction, we investigated specific learning processes and whether these are comparable in younger AN patients to their peers without AN. Findings will contribute to unravelling whether impairments in cognitive processes such as learning are implicated in the development of AN, or whether these impairments are related to chronic starvation of AN. Subsequently, this knowledge will inform treatment directions.

Over the last few years studies have shown that some individuals with AN experience impairments in cognitive functioning. Studies in adults with AN show problems across a wide range of cognitive domains, such as, motor inhibition, visual processing speed, central coherence, visual–spatial ability, attention, learning and memory as well as decision making and cognitive flexibility [9, 10]. Currently it remains unknown however to what extent these difficulties are contributing to the development and maintenance of AN, or in turn, to what extent the chronic underweight of AN causes these cognitive impairments. Although longitudinal studies are the desirable method for answering these questions, these types of studies are costly and hindered by high levels of attrition in AN [11]. An alternative approach to gaining insight into the relation between cognitive functioning and AN is to study patients with a relative short duration of illness (i.e., adolescents) to compare with results in older samples. Seeing that the common age of onset of AN is early to mid-adolescents, studying young people with AN may provide important information about cognitive disturbances in AN at younger age [5].

Interestingly, published data on cognitive functioning in adolescent patients with AN posit a more mixed picture compared to the adult literature. Many studies, commonly including neuropsychological instruments, report no deficits at all or only subtle impairments in e.g. nonverbal intelligence functions and cognitive flexibility impairments such as audiomotor responses, and set-shifting abilities (e.g. [12–18]). In terms of general cognitive functioning, Schilder et al. [19] found that IQ was in fact higher in adolescent AN patients then the norm which suggests a superior cognitive functioning compared to peers.

Looking specifically at learning in AN there is less literature available. One increasingly popular hypothesis, based on recent advances in cognitive neuroscience, posits that persistent AN behaviors may be understood as maladaptive habits, which are driven by abnormal learning processes [20, 21]. This neurobiological “habit model of AN” [22, 23] suggests that for AN patients, eating behaviors become automatic responses very quickly and that little effort is needed to maintain these behaviors. On the other hand, *discontinuing* these dysfunctional habits becomes very difficult, as expressed in the often unsuccessful treatment of AN. In other words, *stimulus-response learning* may be augmented in individuals with AN.

Another important type of learning is category learning, which refers to the ability to make adaptive responses across a wide variety of situations and as such is a fundamental decision making process. Two separate but overlapping learning systems that contribute to category learning are the explicit and implicit learning systems. Explicit learning involves conscious learning, including (sets of) rules and feedback processes (rule-based learning) [24].

On the other hand, implicit learning refers to extracting predictive relationships in the form of statistical regularities or sequence of events from the environment without putting conscious effort into the process or even realizing the learning process at all (procedural-based learning [24]. The two types of learning are related to different brain areas and neural pathways, whereby explicit learning involves the hippocampal and medial temporal areas, whilst implicit learning engages frontal cortico-striatal circuits [25].

Explicit category learning involves both initial acquisition learning and updating explicitly-learned associations. This latter learning aspect is partly determined by a cognitive process called set-shifting, i.e. being able to shift attention between one task and another, whereby poor set-shifting interferes with being able to successfully update these explicitly-learned associations. In recent years set-shifting has gained a lot of attention in AN. While the literature suggests impaired set shifting in adults with AN [26], findings related to set-shifting in adolescent AN samples are mixed and whilst some studies show set-shifting impairments, other studies find that adolescents with AN perform on equal measure to HC groups (for a review see [15]). Mixed findings may be attributable to the complexity of different paradigms assessing different components of set-shifting. One task that has been previously used in studies investigating set-shifting and, more broadly, the ability to update explicitly-learned associations is the Houses and Castle task [27]. Set shifting deficits have been found in individuals with AN and individuals weight restored from AN [18, 28]. Individuals weight restored from AN also displayed hyper-learning, defined as a steeper learning curve, and learned the rules of the task faster than their healthy counterparts. This learning slope, however, was not significantly associated with the shift cost [28]. As far as we know, there are no studies explicitly examining hyper-learning in adolescents with AN. Following the theory that an amplification in learning processes, for example habit learning, is characteristic of AN, arguably then we may expect to find altered learning processes in adolescents with AN. Research on implicit category learning on the other hand is scarce. In fact, to our knowledge only one study looked at implicit learning in adolescents with AN [29]. Firk and colleagues [29] studied an adolescent sample before and after weight gain and found implicit sequence learning, which refers to learning the order of a sequence of stimuli, which is thought to be random, to be impaired, and that this was related to lower BMI. Looking at the adult literature, Shott et al. [30] found that in adults with AN, implicit category learning, which refers to learning how to categorize stimuli according to an unknown and non-verbalizable rule, was impaired. Other studies in adults showed (implicit) attention interferences for food-related words in individuals with patients with AN, but no implicit memory bias [31].

Furthermore, Shott et al. [30] found that implicit category learning was related to heightened novelty-seeking and lower sensitivity to punishment in adults with AN, which hints at the potential association with reward processes. Studies in adults show that the reward-related dopamine system is indeed implicated in cognitive functioning (e.g., reinforcement learning) [32]. Moreover, alterations in dopamine system activity has been associated with depression [33] and anxiety traits [34], both of which are pertinent to AN [34–36]. All of the studies above include adult samples; studies including adolescent samples are scarce. There is some evidence from cerebrospinal fluid and neuroimaging studies that the dopaminergic (DA) system is abnormal in adults ánd adolescents with AN, studies are lacking that directly linked DA function to behavior in AN [37–39]. The DA system is involved in Pavlovian model free learning, as well as habit and goal directed learning [40]. Elevated brain response during reward prediction error tasks may indicate altered Pavlovian stimulus-response learning in AN [38]. However, the interactions between the DA system and learning in AN needs further study. Nevertheless, it has been speculated that plasticity of brain DA function in adolescents is higher than in adults, and that this more flexible DA response may protect from DA-related learning inefficiencies [18]. It is therefore possible that whilst adults with AN display impaired learning, adolescents with AN will have *intact* learning due to age-dependent greater flexibility of their learning circuitry. Alongside the possible influence of the reward system, other potentially important effects on attention-dependents tasks include age-related, motivational and mood-related effects. For example, it has been suggested that those with a relatively short duration of AN (either adult or adolescent) may have ways to compensate poor cognitive functioning by activating more neural activity, or different neural circuits, or both [41]. High levels of perfectionism and fear for making mistakes, often reported by both adults and adolescents with AN [42] may also serve as a compensatory feature contributing to a performance comparable to healthy peers despite perhaps poorer cognitive functioning. With regards to mood disturbances there is evidence suggesting that both depressive and anxious symptomatology contribute to poorer performance on cognitive tasks in adults with AN [43, 44]. Whether depressive and anxious symptomatology contribute to implicit learning in adolescents with AN is another unexplored area. Recent studies have, for instance, indicated that individuals who score high on intolerance of uncertainty perform poorly on threat extinction (as assesses with responses to uncertain auditory stimuli that varied in threat level), which may contribute to suboptimal learning [45]. Intolerance of uncertainty is an important anxiety-related factor and refers to approach and avoidance responses to uncertainty whereby those with higher intolerance levels, are more likely to interpret ambiguous stimuli as more threatening, and have less confidence in their decisions in ambiguous situations [46]. Intolerance of uncertainty has been associated with the reward system [47] and may be an important factor for understanding learning processes in AN seeing that it is pertinent to both adults with AN [48–50] and adolescents [51, 52].

Depression has also been associated with altered learning and specifically in reward related context [53]. Our understanding of what brain regions and neurotransmitter systems are involved is still limited and again studies including adolescent samples are lacking However, several factors could play a role. Anxiety and depression as well as AN are associated with elevated cortisol levels as a sign of high stress, which could interfere with cognitive flexibility and learning [54]. Stress has been found to alter dopamine and noradrenaline circuitry and thereby altering working memory function and learning [55]. In sum, whilst there is some evidence for altered learning processes in adults with AN, and associations with affective states, studies investigating learning processes in adolescents with AN are scarce. Building on the theory that altered learning processes are characteristic of AN, we expect to find these alterations in adolescents as well.

### Aims & hypotheses

The aims of this study were twofold. We wanted to test the hypothesis that implicit learning is intact in adolescent AN, similarly to explicit learning studies, as this may provide insight into the development of cognitive functioning from childhood years to adulthood and may shed some light onto the relation between learning abnormalities and the (long-term) neurobiological starvation effects in AN. Second, we wanted to test whether depressive and anxiety symptoms are related to worse learning performance in adolescents with AN.

## Method

### Participants

A total of 90 adolescent participants (11–17 years old) were recruited from two different sites (the Netherlands - NL, United States of America - USA), which will be described here separately. No participants were excluded in the USA groups; 3 participants were excluded in the NL groups.

#### NL sample

Twenty adolescents with a current diagnosis of AN according to DSM 5 criteria were recruited from a Dutch specialized Eating Disorders center (AN-NL group). Diagnoses were established by psychiatrists or clinical psychologists and confirmed with the Eating Disorder Examination (EDE [56];). Participants were excluded in the case of alcohol and drug abuse, history of or current diagnoses of other psychiatric disorders such as dementia, schizophrenia or mental retardation, and current diagnoses of diabetes, or a neurological disorder. Of these 20, 6 were taking anti-psychotics and 1 was taking mood-stabilizers. None were taking anti-depressants or sedatives. Eighteen healthy control adolescents were recruited in the Utrecht (NL) area through local advertisement flyers posted in a number of high schools, sports clubs and community centers (HC-NL group). They were included if they had no history of neurological medical diagnoses that may affect cognitive functioning, and no first-line relatives with a diagnosis of an eating disorder. Before participation, the experimenter completed the Mini International Neuropsychiatric Interview (M.I.N.I.: [57]; Dutch version: [58]), in order to screen for any possible (undiagnosed) psychiatric disorders. If there was any indication of an (undiagnosed) disorder (as seen from any of the subsections of the M.I.N.I.) participants were excluded from the study (*n* = 3).

#### USA sample

Twenty-six adolescents with a diagnosis of AN (AN-USA group) were recruited through an Eating Disorders program at a children’s hospital and a specialized Eating Disorder center (USA). All participants met DSM 5 criteria for AN (*n* = 24) or broadly defined AN (restricting atypical AN, *n* = 2) at the time of enrolment of the study. All individuals with AN completed the Clinical Diagnostic Interview Schedule for Children 4.0, to assess all major psychiatric diagnoses [59]. Participants were excluded if there was any indication of current substance use or other psychiatric disorders including dementia, mental retardation, schizophrenia or any neurological disorder. Those with diagnoses of anxiety and depression were included. Of these 26, 10 were taking anti-depressants and 4 were taking anti-psychotics. None were taking mood-stabilizers or sedatives. Twenty-six adolescent non-AN controls (HC-USA group) were recruited through local advertisements in the Denver metropolitan areas (USA). They completed the Clinical Diagnostic Interview Schedule for Children 4.0, to rule out any current or previous psychiatric disorders [59]. Non-AN controls had a lifetime history of body weight between 90 and 110% of ideal body weight since menarche.

### Clinical measures

The NL-AN group’s BMI was assessed before participation, by measuring weight on a digital Tanita scale (Tanita Cooperation of America, Inc., Arlington Heights, IL) and height with a stadiometer. The NL-HC group’s BMI was assessed by asking participants to state their height and weight. All BMI were then calculated as kg/m^2^.

The USA-AN group’s BMI (kg/m^2^) was obtained from their hospital chart (weight was measured on a digital scale daily). The weight date was on the day of the testing session, which was between 1 and 2 weeks into treatment. The USA-HC group’s BMI (kg/m^2^) was assessed immediately before the testing session by weighing them on a digital Detecto scale (Detecto, Webb City, Missouri) and measuring their height with a Seca stadiometer.

All four groups of participants (NL-AN, NL-HC, USA-AN and USA-HC) were asked to fill out two questionnaires; the Children’s Depression Inventory to measure possible depressive symptoms (CDI: [60], α = 0.92; Dutch version: [61], α = 0.88 and a scale measuring intolerance of uncertainty (IUS: [62], α = 0.96; Dutch version: [63], α = 0.93). We used the Intolerance of Uncertainty Scale (IUS) to assess symptoms of anxious pathology. Intolerance of uncertainty is a key component of anxiety, and a wealth of evidence shows the contribution of intolerance of uncertainty to anxiety across a wide range of anxiety disorders and other psychological disorders (see a recent review by [64]).

### Implicit category learning task

**Fig. 1:**
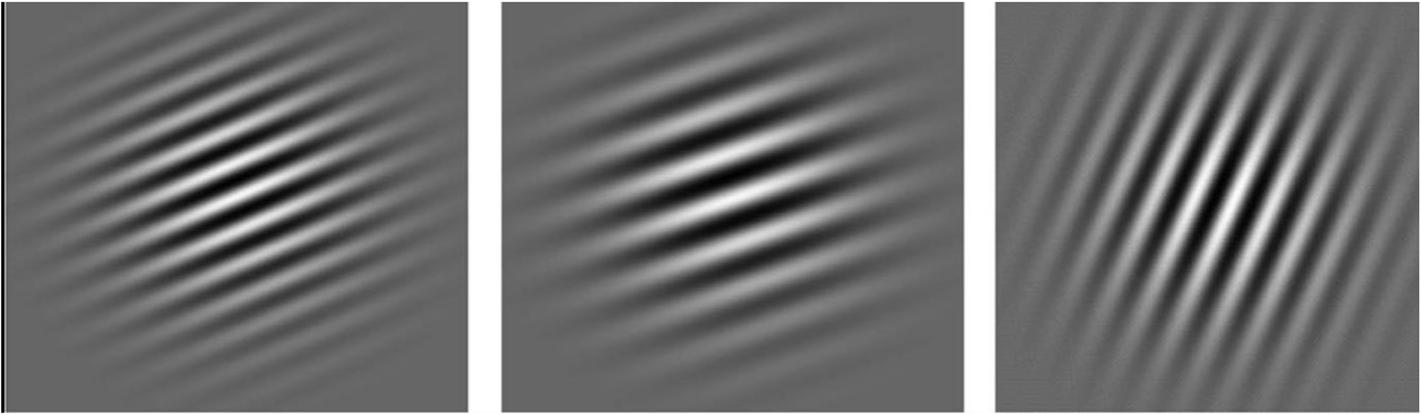
Examples of Gabor patch stimuli, image taken from Shott et al. [30]

All 908 participants were asked to do an implicit category learning task, as previously used in Shott et al. [30]. In this task, participants were presented with Gabor patches (see Fig. 1 for examples), which they were asked to categorize into one of two categories (A and B). Each Gabor patch was presented until the participant’s response was made (“z” and “/” keys for categories A and B, respectively). After this, the participant received feedback for 1 s: the screen displayed the words “correct” or “wrong” respectively. Immediately after 1 s of feedback, a mask was displayed for 5 s in order to prevent participants from responding to the after-image of the previous stimulus. Then, the next trial began. The rule, unknown to participants, by which the Gabor patches had to be categorized, required a linear integration of two stimulus dimensions (spatial frequency and orientation of the lines in the stimulus). In each testing session, each of the presented stimuli was unique in its combined spatial frequency and orientation dimensions. For each testing session there were 80 trials, for which an equal number of Gabor patches from category A and B were generated randomly by sampling from two bivariate normal distributions (as originally done by [65]). Each Gabor patch was generated using MATLAB routines from Brainard’s [66] Psychophysics Toolbox.

The NL groups performed this task on a computer with a 15.4″ screen with a 1680 × 1050 resolution. The Gabor patches were thereby approximately 5 cm in height and at an approximate viewing distance of 45 cm, they subtended a visual angle of about 6.4°. The USA groups performed this task on a computer with a 21″ screen with a 1360 × 1024 resolution. Each Gabor patch was thereby 7 cm in diameter, which subtended a visual angle of about 8.9° from an approximate viewing distance of 45 cm.

This paradigm has been used extensively to gain a better understanding of the underlying processes in category learning in both normal and patient populations [67, 68].

### Explicit category learning task

**Fig. 2:**
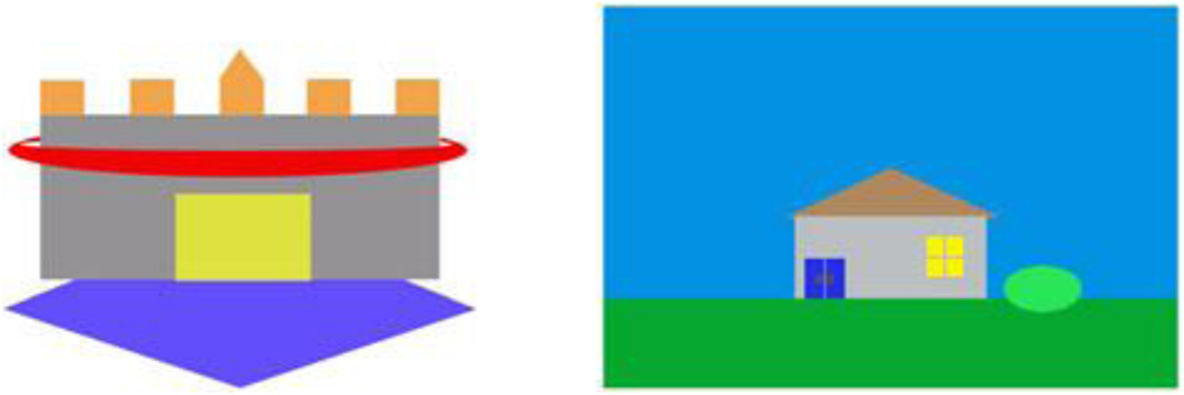
Examples of castle and house stimuli, image taken from Shott et al. [18]

A selection of our sample (AN-NL and HC-NL; total *n* = 38) additionally completed an explicit category learning task, in addition to the implicit category learning task, i.e. the Houses & Castles task [18]. In this task, participants were randomly categorized into two groups: Houses group and Castles group. In each trial, participants were presented with either a cartoon image of a house or a castle (see Fig. 2 for examples), depending on their group, which was presented until the participant made a response. Each stimulus belonged to a category (“A” or “B”) based on an unknown rule. Participants were asked to categorize the stimuli by pressing a key (“z” key and “/” key for categories “A” and “B” respectively). Immediately after the participant’s response, feedback was shown for 0.75 s: displaying either the word “correct” or “wrong” beneath the image of the stimulus. This was followed by 1 s of blank screen, after which the next trial began. There was a total of 160 trials. Four dimensions with binary values could differ per stimulus per trial: castle stimuli – shape of foundation (diamond or square), location of ramparts (above or sunken into walls), number of rings around castle (one or two), color of drawbridge (yellow or green); house stimuli – color of door (red or blue), lighting inside window (light on or off), shape of roof (flat or triangular), type of plant (shrub or tree). During the first 80 trials, the rules for categorization were as follows: castle stimuli – shape of foundation (category “A”: square, category “B”: diamond); house stimuli – shape of roof (category “A”: flat, category “B”: triangular). During the last 80 trials, the rules for categorization were as follows: castle stimuli – number of rings (category “A”: one, category “B”: two), house stimuli – type of plant (category “A”: tree, category “B”: shrub). Participants were never informed of the rule shift and had to infer all rules from the provided feedback. Participants were given feedback on every trial and the contingencies were the same in each trial.

This task has been used many times before to reliably test set shifting/explicit and implicit category learning across normal and patient populations [18, 27].

Participants performed this task on a computer with a 15.4″ screen with a 1680 × 1050 resolution. Stimuli were approximately 6.5 cm in height and at an approximate viewing distance of 45 cm, they subtended a visual angle of about 8.3°. The stimuli were generated using MATLAB routines from Brainard’s [66] Psychophysics Toolbox.

### Procedure

This study was approved by both appropriate USA and Dutch (medical) ethical committees. All participants and their parents or legal guardians gave consent for participation in this study. USA data was collected between February 2012–February 2014. An additional Dutch sample was included between October 2014 and August 2016 in order to 1) increase power for the implicit learning task; and 2) build on the [18] study by analyzing associations between the explicit learning processes and intolerance of uncertainty.

Primary analyses included a between-subjects design, where all participants were asked to do an implicit category learning task, and a sub-group of participants (NL-AN and NL-HC groups only) were asked to additionally do an explicit learning task.

In the case of participants who were not administered the additional explicit learning task (USA-AN and USA-HC groups), they were asked to complete the implicit category learning task at the beginning of the testing session. In the case of the other sub-group of participants (NL-AN and NL-HC groups), they were asked to first perform the explicit learning task, followed by the implicit learning task after a small break. This order was chosen as the explicit learning task is the easier one of the two so we expected that participants would thereby stay motivated enough after the first task to complete the second task.

All participants were asked to fill out all questionnaires at the end of the testing session. The experimenter stayed with the participant at all times during the testing session in case of fatigue, questions about the tasks or questionnaires, or in case of early termination of the experiment. Participants were not given compensation for their participation.

### Statistical analyses

Statistical Package for the Social Sciences version 26 was used for the analyses. In order to see whether there were any significant differences in age or BMI between the AN adolescents and the non-AN controls, and between the USA and NL groups, independent samples t-tests were run with Bonferroni corrections for multiple testing. Sphericity as well as homogeneity of variances were checked and corrected for accordingly at all times. Where sphericity could not be assumed within an ANOVA, the Greenhouse-Geisser results are reported. Estimates of effect size are calculated using partial eta squares or Cohen’s D [69], where .2 = small effect, .5 = medium effect and .8 = large effect.

### Learning outcomes

#### Model-based analyses implicit learning task

Using mathematical models, this task allows for insight into the specific approach participant use when learning the task [67, 70, 71]. As explained by Shott et al. [30], these models can identify AN patients who adopted a procedural-based approach to learning compared to healthy controls, in order to assess impairments in procedural-based learning in patients. Two classes of models will be compared, namely the *procedural-based* (PB) approach, and the *hypothesis-testing* (HT) approach. The optimal PB model assumes that participant used the rule displayed in Fig. 1 as the solid line. The second PB model was the general linear classifier (GLC), which also assumes that the participant’s decision on each trial is based on a linear integration. HT models assume that the participant set a criterion and that there were four response regions: low frequency/ shallow angle, low frequency/steep angle, high frequency/shallow angle and high frequency/ steep angle (for a detailed explanation of these models see Shott et al. [30].

#### Statistical analyses

Following procedures as described by Shott et al. [30], to explore differences in implicit learning performance, a 2 (group) × 4 (block) mixed-design ANOVA (to compare AN to HC) was run with the following measures: 1) accuracy (number of correct responses divided by number of trials), 2) reaction time (RT, in seconds) and 3) reaction time variability (standard deviation of reaction time). Moreover, 4) a learning curve (accuracy in block 4 – accuracy in block 1) was computed and 4-way ANOVA’S were used to examine group differences. Post-hoc tests were examined to ensure no country group differences (NL versus USA AN groups).

Following procedures from Shott et al. [30], for the explicit learning task, to explore differences in accuracy (number of correct responses divided by the number of trials) in the explicit learning task, a 2 (group) × 8 (block) mixed-design ANOVA was run (in the Dutch samples only).

### Learning outcomes and anxious and depressive symptomatology

To explore associations between implicit learning outcomes and anxious and depressive symptoms Pearson’s correlation analyses were run in all four groups including, depression, BMI age, and learning curve (implicit learning) variables (for an elaborate explanation on the using the learning curve within the correlation analyses, rather than the other outcomes we refer to procedures described by Shott & colleagues [18].

To explore associations between implicit learning outcomes, explicit learning, anxious and depressive symptoms, Pearson’s correlation analyses were run in AN (NL-AN only) and in HC (NL-HC only) separately including learning curve (implicit learning outcome), accuracy block 5 (explicit learning), intolerance of uncertainty, depression, BMI and age.

## Results

### Age and BMI

There were no significant difference in age between AN patients (NL versus USA (NL: mean = 15.60, SD =1.23; USA: mean = 14.73, SD = 1.56)) and non-AN controls (NL versus USA (NL: mean = 15.22, SD = 1.47; USA: mean 14.19, SD: 1.86)). There was a significant difference in age between the two USA and the NL groups whereby the USA groups were slightly younger than the AN-NL group (*t*(86.34) = − 3.73, *p* < 0.01, Cohen’s *d* = 0.79), but including age as a covariate did not change the results.

As expected a significant difference in BMI between ANs and HCs was found (see Table 1). Low BMI is inherent to AN diagnosis and was therefore not added into the analyses as covariate. No significant differences in BMI between the two AN groups or between the two HC groups were found.

**Table 1:** Age, BMI, depression, intolerance of uncertainty, implicit leaning (means, standard deviations and ranges; *N* = 90)

Measure	AN-USA *N* = 26	AN-NL *N* = 20	HC-USA *N* = 26	HC-NL *N* = 18	Test statistic ANOVA
Age ** ^a^	*M* = 14.73 *SD* = 1.56 Range = 12–17	*M* = 15.60 *SD* = 1.23 Range = 13–17	*M* = 14.19 *SD* = 1.86 Range = 11–17	*M* = 15.22 *SD* = 1.47 Range = 12–17	*F*(3,85) = 5.61 *p* < 0.01 η^2^ = 0.17
BMI ** ^b c^	*M* = 16.14 *SD* = 1.53 Range = 12.49–18.34	*M* = 17.28 *SD* = 1.82 Range = 13.30–20.07	*M* = 20.21 *SD* = 2.45 Range = 16.41–25.88	*M* = 20.37 *SD* = 2.53 Range = 15.82–24.40	*F*(3,85) = 23.56 *p* < 0.01 η^2^ = 0.45
CDI ** ^a b c^	*M* = 12.31 *SD* = 9.00 Range = 0–28	*M* = 23.89 *SD* = 2.23 Range = 20–27	*M* = 3.04 *SD* = 2.72 Range = 0–9	*M* = 7.56 *SD* = 5.44 Range = 2–22	*F*(3,85) = 51.23 *p* < 0.01 η^2^ = 0.64
IUS ** ^b d^	*M* = 71.31 *SD* = 20.55 Range = 35–107	*M* = 80.53 *SD* = 14.55 Range = 41–107	*M* = 48.27 *SD* = 18.09 Range = 27–104	*M* = 64.83 *SD* = 15.69 Range = 28–92	*F*(3,85) = 13.66 *p* < 0.01 η^2^ = 0.33
Implicit learning accuracy (log) * ^c^	*M* = −.21 *SD* = .07 Range = −.33 - -.08	*M* = −.18 *SD* = .07 Range = −.33 - -.07	*M* = −.21 *SD* = .06 Range = −.34 - -.14	*M* = −.27 *SD* = .05 Range = −.34 - -.14	*F*(3,85) = 3.28 *p* = 0.02 η_p_^2^ = 0.10
Implicit learning reaction time (log)	*M* = .09 *SD* = .15 Range = −.18–.49	*M* = .04 *SD* = .11 Range = −.15–.29	*M* = .14 *SD* = .14 Range = −.11 - -.42	*M* = .06 *SD* = .17 Range = −.28–.30	*F*(3,85) = 2.18 *p* = 0.10
Implicit learning reaction time variability (log)	*M* = −.15 *SD* = .24 Range = −.63–0.31	*M* = −.18 *SD* = .19 Range = −.60–.15	*M* = −.03 *SD* = .31 Range = −.57–.58	*M* = −.05 *SD* = .24 Range = −.51–.45	*F*(3,85) = 1.74 *p* = 0.16
Implicit learning curve (accuracy in last block minus accuracy in first block)	*M* = .10 *SD* = .12 Range = −.11–.31	*M* = .03 *SD* = .10 Range = −.13–.2	*M* = .09 *SD* = .12 Range = −.11–.31	*M* = .04 *SD* = .10 Range = −.13–.20	*F*(3,85) = 1.92 *p* = 0.13

*CDI* Children’s Depression Inventory, *IUS* Intolerance of Uncertainty

* = *p* < 0.05; ** = *p* < 0.01

^a^AN-USA and AN-NL differ significantly

^b^AN-USA and HC-USA differ significantly

^c^AN-NL and HC-NL differ significantly

^d^HC-USA and HC-NL differ significantly

### Implicit learning task

Due to the mixed design ANOVA on implicit learning data showing heterogeneity of variance on all measures (i.e. non-normal distribution), all data was logarithmically transformed to normalize the data and reduce heterogeneity of variances (according to [72]). A skewness analysis of the untransformed data showed that implicit learning data was indeed skewed (max skewness value = 4.78, *SES* = 0.25). The skewness observed in the logarithmically transformed data was improved as compared to the untransformed data, with all skewness values lying between − 0.54 and 0.36 (*SES* = 0.25), which is within the acceptable range of skewness (e.g. [73]). We therefore deemed the logarithmic transformation adequate to normalize the data. Another 2 (group) × 4 (block) mixed-design ANOVA was then run on the log10 transformations of accuracy, reaction time and reaction time variability. For a summary of all implicit learning task results, see Table 1.

#### Implicit learning task: accuracy

**Fig. 3:**
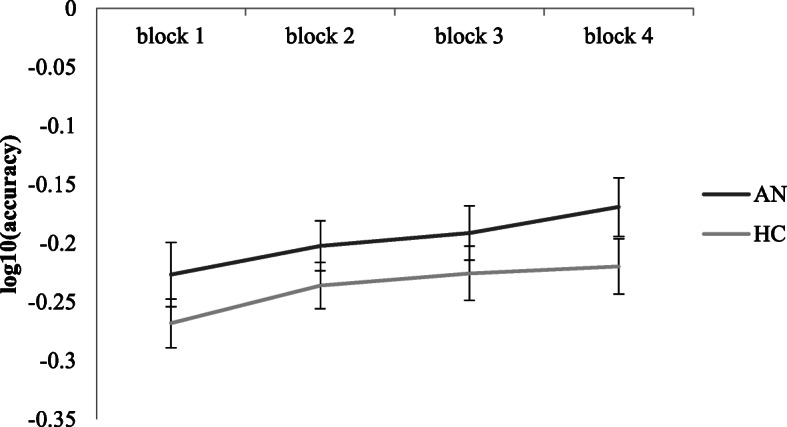
Changes in accuracy (number of correct responses divided by the number of trials, log transformed) in implicit learning task across blocks, differentially between AN HC groups. Error bars show standard error

The ANOVA revealed a main effect of group, *F*(1,88) = 7.77, *p* = 0.01, *η*_*p*_^*2*^ = 0.08, where the AN groups were overall more accurate than the HC groups (small effect). A main effect of block was found, *F*(2.30,201.92^1^) = 20.59, *p* < 0.01, *η*_*p*_^*2*^ = 0.19, where all groups improved across blocks. No significant interaction of block x group was found. For an illustration of the accuracy results, see Fig. 3*.* The NL and USA AN groups did not differ significantly from each other.

#### Implicit learning task: reaction time

No significant main effect of group on reaction time was found. There was a significant main effect of block on reaction time, *F*(2.58,227.17) = 28.54, *p* < 0.01, *η*_*p*_^*2*^ = 0.25, where participants’ reaction times decreased across blocks. No significant group x block interaction was found. The NL and USA AN groups did not differ significantly from each other.

#### Implicit learning task: reaction time variability

**Fig. 4:**
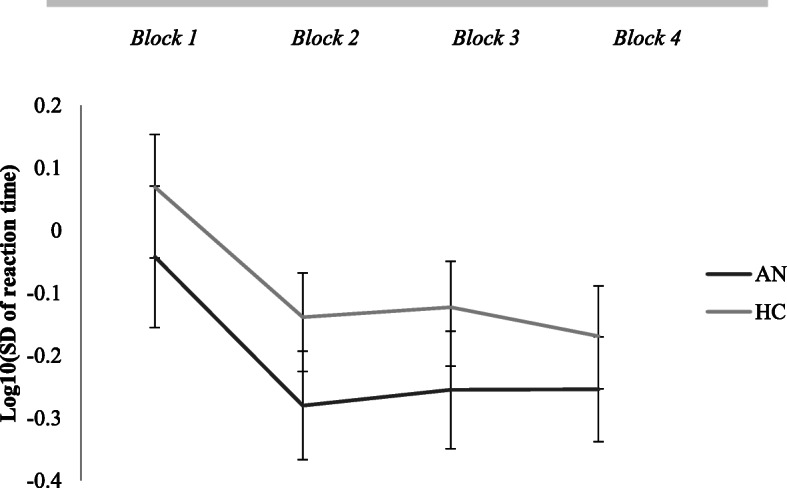
Changes in reaction time variability (standard deviation of reaction time in seconds, log transformed) in implicit learning task across blocks, differentially for AN and HC group

For the reaction time variability, a significant main effect of group was found, F(1,88) = 5.51, *p* = 0.02, *η*_*p*_^*2*^ = 0.06, where the AN group showed less variability in reaction times than the HC group. A significant main effect of block was found, F(2.31,203.22) = 25.32, *p* < 0.01, *η*_*p*_^*2*^ = 0.22, where all participants’ variability decreased over time. No significant block x group interaction was found. The reaction time variability results are displayed in Fig. 4. The NL and USA AN groups did not differ significantly from each other.

#### Implicit learning task: learning curve

No significant differences in learning curve (accuracy in last block minus accuracy in first block) between the AN and HC groups were found. The NL and USA AN groups also did not differ significantly from each other.

#### Implicit learning task: model results

**Fig. 5:**
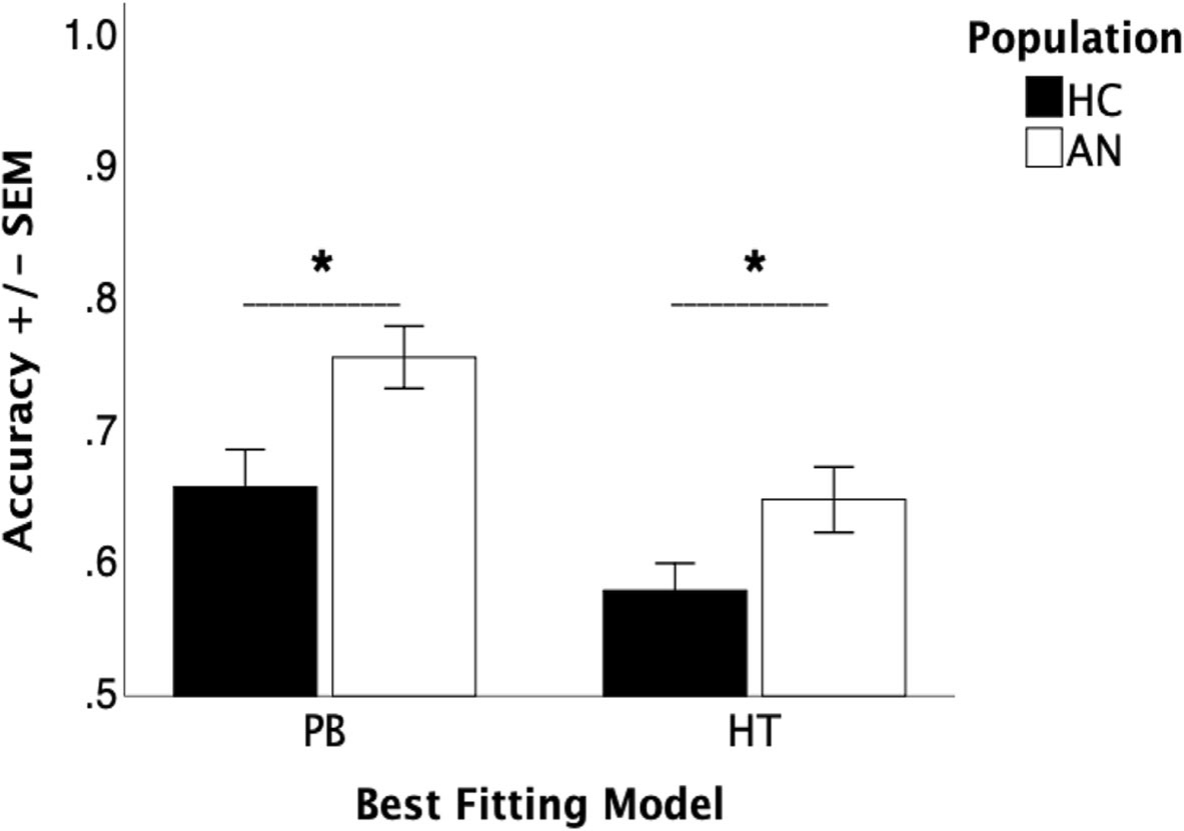
Accuracy results for the Hypothesis Tested (HT) versus Procedural Based (PB) method of learning (*p < .05)

In line with Shott et al. [30], to determine whether the model-based subgroups differed, accuracy rates in the final block for the AN and HC participants who used either *procedural-based* (PB) or *hypothesis-testing* (HT) approach were contrasted (see Fig. 5). T-tests showed that for both the AN and HC participants accuracy for the PB approach was significantly better compared to the HT approach (AN: *p* < .01; HC: *p* < .05). Moreover, for both the PB and HT approach, the AN participants performed more accurately than the HC participants (*p* < .01, when controlling for depression, anxiety or medication *p* < .05).

### Explicit learning task

No heterogeneity of variance was found for the planned ANOVA, therefore no transformation had to be performed on the set-shifting data. For a summary of explicit learning task results, see Table 2.

**Table 2:** Means, standard deviations and ranges of explicit learning task results in the NL groups (*N* = 38)

Statistic	AN *N* = 20	HC *N* = 18	Test statistic t-test
Set-shifting overall accuracy**	*M =* 0.86 *SD =* 0.21 Range *=* 0.25–1.00	*M =* 0.80 *SD =* 0.15 Range *=* 0.46–0.99	*t*(36) = − 3.37 *p* < 0.01 *d =* 1.10
Set-shifting accuracy block 4*	*M =* 0.90 *SD =* 0.21 Range *=* 0.25–1.00	*M =* 0.83 *SD =* 0.24 Range *=* 0.25–1.00	*t*(36) = − 2.13 *p* = 0.04 *d* = 0.69
Set-shifting accuracy block 5*	*M =* 0.73 *SD =* 0.15 Range *=* 0.25–0.95	*M =* 0.67 *SD =* 0.17 Range *=* 0.25–0.95	*t*(36) = − 2.76 *p* < 0.01 *d* = 0.90
Shift cost (accuracy block 5 minus accuracy block 4)	*M =* 0.18 *SD =* 0.24 Range *=* − 0.45-0.75	*M =* 0.18 *SD =* 0.25 Range *=* − 0.45-0.75	*t*(36) = 0.23 *p* = 0.82 *d* = 0.07

* = *p* < 0.05; ** = *p* < 0.01

### Explicit learning: accuracy

**Fig. 6:**
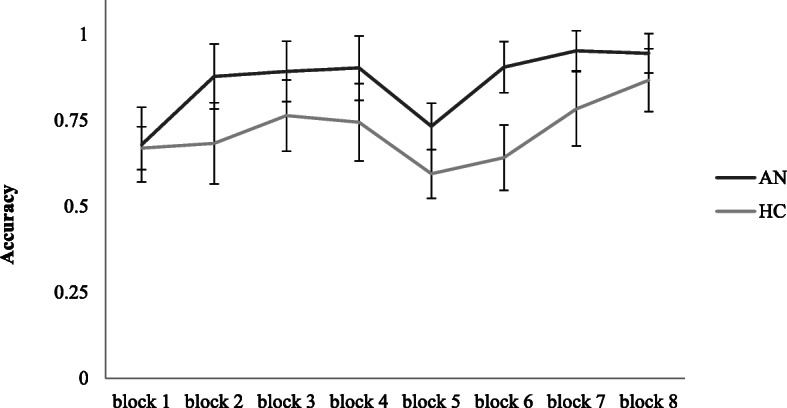
Changes in accuracy (number of correct responses divided by number of trials) in the explicit learning task across blocks, differentially for both groups. Error bars show standard error

The ANOVA revealed a significant main effect of group, F(1,36) = 11.35, *p* = 0.01, η_p_^2^ = 0.24, where the AN group was consistently more accurate than the HC group (large effect). A significant main effect of block was found, F(3.39,122.02) = 10.94, *p* < 0.01, η_p_^2^ = 0.23. No significant block x group interaction was revealed. For a visualization of the explicit learning task accuracy results, see Fig. 6.

### Explicit learning: shift costs

To explore whether there were any differences in shift cost (accuracy in block 5 minus accuracy in block 4) between the two groups, an independent samples t-test was run. To determine the impact of the actual shift, a Shift-Cost score was computed by subtracting each participant’s accuracy on block 5 from their accuracy on block 4 (higher scores equaled a greater shift-cost). Group differences indicate how well a particular group did, compared to the other, at coping with the rule change. No significant differences were found. To investigate this further, an independent samples t-test was run on accuracy in block 5, as well as accuracy in block 4, between groups. After Bonferroni corrections, it was found that accuracy in block 5 differed between groups, t(36) = 2.76, *p* = 0.02, Cohen’s d = 0.90, but not accuracy in block 4 (this only yielded a significant result before Bonferroni corrections, t(36) = 2.13, *p* = 0.04, Cohen’s d = 0.69). This suggests that participants were nearly as good as each other at the end of block 4, *but dealt with the set change after the rule shift differently, yielding different accuracies in block 5.*

### Relationships between implicit learning, intolerance of uncertainty, depression, BMI and age

As there were significant differences between the USA and NL groups regarding the Intolerance of Uncertainty Scale and Children’s Depression Inventory in both the HC and AN groups 4 different analyses were run including learning curve, intolerance of uncertainty, depression age and BMI. In the USA-AN, NL-AN and NL-HC groups no significant correlations were found between any of the implicit learning outcomes and the clinical variables. In the USA-HC group a smaller learning curve was associated to a higher BMI (*r* = −.47*, p =* 0.02) and to a higher age (*r* = −.43, *p* = 0.03).

Relationships between implicit learning, intolerance of uncertainty, depression, BMI, age and explicit learning (NL groups only).

In the NL-AN group, IUS scores were significantly associated to explicit learning outcomes (Accuracy: *r* = .49*, p =* 0*.*03; Costs: *r* = −.46, *p =* 0.05), whereby higher intolerance of uncertainty was associated to higher SS accuracy and lower SS costs (i.e. more/stronger intolerance of uncertainty was related to better learning).

## Discussion

In this study we aimed to understand implicit and explicit learning in adolescents with AN, and to explore associations between learning outcomes and anxious and depressive symptomatology. Interestingly, in terms of implicit learning, accuracy performance of AN participants was superior to that of the HC, and this was true for both the *procedural-based* (PB) and the *hypothesis-testing* (HT) model types. As expected, performance on the other implicit learning outcomes, reaction time and variability in these times, were comparable between the AN and HC participants. Similarly, for explicit learning, AN participants had higher accuracy rates compared to HCs. In both the combined AN and HC groups there were no associations between the implicit learning outcomes and clinical variables such as age, BMI, intolerance of uncertainty and depression. In the USA-HC group poorer implicit learning was associated to lower BMI and lower age, which may be due to developmental processes.

The finding of superior accuracy outcomes in AN on both tasks applied in this study is particularly interesting, seeing that a recent systematic review by Olivo et al. [74] concludes that on most cognitive domains, adolescents with AN are *comparable* to their peers in term of behavioral performance. Our finding of better performance compared to non-AN controls on a specific type of learning task adds new important information regarding cognitive functioning in adolescents with AN. Looking at learning more broadly, findings fit with the theory that individuals with AN may have augmented *stimulus-response learning*. As posited by the “habit model of AN” ([20]; Walsh, 2013), for individuals with AN, behaviors become automatic responses quickly and are then also maintained without much effort. Discontinuing these habits however is much harder, which may explain why some AN symptoms remain difficult to treat.

Furthermore, an earlier study showed that patients with AN had higher IQs than the population norm, which may also contribute to better performance [19]. Another possible explanation for more optimal behavioral performance may lie with high levels of perfectionism that may partly drive this overperformance [75]. That is, adolescents who develop AN put in more effort to “get it right”, which would reflect the high perfectionism commonly present in individuals with AN [42]. A limitation of the study however is that it did not include IQ or perfectionism measures so this remains speculation. Future studies on learning in AN should apply perfectionism scales and test this hypothesis.

It is also possible that adolescents with AN, with a (usually) shorter duration of illness are in a state of cognitive and perfectionistic overdrive, driven by a brain pathophysiology that is in a state of overexcitability and associated with high intellectual capacity. Interestingly, such an “overexcitable cognitive ability” has been associated with hyper-reactivity of the central nervous system [76], which is associated to a risk for psychopathology [77]. An important neural system implicated in cognitive functioning and perhaps explanatory for our results of better learning performance in adolescent AN is the dopamine system. Striatal dopamine pathways are involved in major cognitive domains such as feedback sensitivity, which in turn affects learning processes. Indeed, previous literature highlights alterations in feedback sensitivity, especially punishment sensitivity, in adults and adolescents with AN [37, 78] which may affect learning strategies [32]. Furthermore, brain dopamine circuitry is a major contributor to model free and model based learning, namely Pavlovian prediction error learning, habit learning and goal directed instrumental learning [40]. This study was not designed to test dopamine circuit function and thus does not allow testing for these hypotheses. We are currently planning future studies that will include biological markers of the dopamine system when investigating learning in AN.

Our results are in line with previous literature showing that cognitive processes that may be disturbed in adults with AN are intact in adolescents with AN [12, 13, 15, 16, 18, 30]. It is therefore possible that cognitive deficits in adults are at least partly contributable to the illness itself, which may be explained by the neurobiological effects of long-term starvation [79]. The initial hyper-drive in adolescent AN during the continuing duration of illness (and associated long-term malnourishment) then transfers into a state of burn-out (and associated cognitive problems) in adults with long-term AN (as theorized by [74, 80]). Indeed, Shott et al. [30] found that adults with AN still performed poorly on the same implicit learning task used in this study, even if they applied the correct model or strategy. Whether these suspected illness-related changes are permanent is an important question for further research to examine, yet the relation between cognitive functioning and malnourishment in AN is complex, and study findings aiming to entangle this relationship are mixed. For example, as highlighted by a recent review [81], whilst some literature suggests that cognitive difficulties in AN are related to low weight, other studies find that cognitive deficits were not or only partly associated with malnourishment in AN (see also [82]). Also data on the reversibility of these potential malnourishment-related cognitive deficits is mixed; some studies find that AN-related cognitive difficulties are reversable after weight gain [83], whilst others find that even short-term malnourishment can lead to irreversible brain changes in adolescents with AN (for a review see [84]). The lack of associations between learning outcomes and clinical variables in the AN group was unexpected, in particular seeing that some recent studies demonstrated negative effects of anxious and depressive symptoms on cognitive functioning in AN (i.e. social problem solving; [85]) and central coherence [86]. Due to group differences in scores on the clinical instruments analyses were run in the separate smaller groups. This may have resulted in a power issue which in turn may have resulted in non-significant associations that in fact could be significant with a larger sample. On the other hand, a recent meta-analysis concluded that depression is not associated to set-shifting in adults [10]. In line with this finding, a recent study including adolescents showed that despite higher levels of depression in the AN group, set-shifting ability did not differ between AN and healthy controls [16]. Of note, results of the current study show that intolerance of uncertainty was significantly higher in the AN adolescents than in the non-AN controls, and that stronger intolerance of uncertainty was related to better explicit learning confirming that intolerance of uncertainty may be an important clinical factor in adolescents with AN [52]. How and to what extent intolerance of uncertainty fits into AN pathology requires further examination in future studies. Whilst no differences between the NL and USA groups on learning outcomes were found, interestingly, the NL and USA groups did differ in terms of anxious and depressive symptomology, with the NL-AN group reporting more severe depression and intolerance of uncertainty. Whether this is indeed a cultural difference in terms of severity, or can be explained by other cultural differences (i.e. interpretation of questions) we can’t conclude from this study.

Whilst these results are promising, it is important to keep in mind that the observed effects are small and that due to relatively small groups, interpretation should be done with caution and replication studies are warranted, in particular the findings for the explicit learning task seeing that only the NL groups completed these (18 HC versus 20 AN = total *n* = 38). However, for the associations between learning outcomes and clinical variables, some correlation coefficients were in fact quite high, suggesting that in larger groups significant correlations may be detected. Further studies should include larger samples and it is recommendable to record (clinical characteristics of) those who were invited but refused to take part in order to avoid any participation biases. Future studies may also want to ensure matching the HC group to the patient groups to optimize comparability.

Although we did include age in this study, we did not include illness duration, which may well be an important factor seeing the effects of more chronic and long-term AN on the brain, and cognitive processes. Lastly, due to a technical error we had to rely on self-report weight data for some participants, future studies should ensure objective weight information. Moreover, studies aiming to understand the complex relation between BMI and cognitive processes in adolescents may benefit from using the WHO values (% median BMI).

## Conclusions

Taken together these findings shed light on learning processes in adolescents with AN, in that learning appears intact, or even enhanced, compared to their peers. This may be an indication that cognitive difficulties such as impaired implicit and explicit learning in adults with AN may result from (enduring) starvation or other illness related factors. Future research should aim to examine the effects of acute and long-term malnourishment and weight gain on learning processes.. The better performance in the adolescent AN group is in line with other research examining cognitive functions in adolescents, and the opposite compared to research in adults. It is therefore possible that the brain activation in AN when young is in a form of hyper-learning, i.e. showing steeper learning curves then their peers without AN, and that this hyper-learning is in turn potentially driven by anxiety and perfectionism to result in excellent task performance [76, 77]. However, this state is not sustainable, food restriction may also take its toll on the brain, and this may eventually lead to poor performance in adults.

Taking into account studies that do find learning impairments in adolescents with AN, further research should focus on unravelling different learning processes and their underlying neurocircuits. This may have direct clinical implications in that identifying the underlying pathophysiology of altered category learning in AN may allow us to identify interventions that maintain normal cognitive flexibility from young to adult age, which could improve outcome when treating adults with AN.

## Data Availability

The datasets used and/or analysed during the current study are available from the corresponding author on reasonable request.

## Abbreviations

AN: Anorexia nervosa
APA: American Psychology Association
BMI: Body mass index
CDI: Children’s Depression Inventory
DA system: Dopaminergic system
DSM-IV: Diagnostic statistical manual-IV
EDE: Eating Disorder Examination
HC: Healthy control
HT approach: Hypothesis-testing approach
IQ: Intelligence quotient
NL: Netherlands
IUS: Intolerance of Uncertainty Scale
PB approach: Procedural-based approach

## Footnotes

1 As Mauchly’s test of sphericity was violated Greenhouse-Geisser corrected values are reported
